# Intact *pks15/1* in Non–W-Beijing *Mycobacterium tuberculosis* Isolates

**DOI:** 10.3201/eid1205.051208

**Published:** 2006-05

**Authors:** Angkana Chaiprasert, Jutaporn Yorsangsukkamol, Therdsak Prammananan, Prasit Palittapongarnpim, Manoon Leechawengwong, Chertsak Dhiraputra

**Affiliations:** * Mahidol University, Bangkok, Thailand;; † Drug-resistant TB Fund, Bangkok, Thailand;; ‡ National Center for Genetic Engineering and Biotechnology, Pathumthani, Thailand;; § Vichaiyut Hospital, Bangkok, Thailand

**Keywords:** 1*pks15/1*, W-Beijing, tuberculosis, PGL, Mycobacterium

## Abstract

To determine whether intact *pks15/1* is unique to the W-Beijing family, we investigated 147 *Mycobacterium tuberculosis* strains with different IS*6110* genotypes. Intact *pks15/1* was found in 87.8% of cerebrospinal fluid and 84.9% of sputum isolates. It was found not only in W-Beijing strains (≈97%) but also in other genotypes (38.5%–100%).

Two structurally related families of cell envelope lipids, phthiocerol diesters and phenolic glycolipids, are virulence factors of *Mycobacterium tuberculosis* and *M. leprae*. They are also produced by other slow-growing species, in particular the pathogenic species *M. marinum*, *M. ulcerans*, and members of *M. tuberculosis* complex (*1*). Phthiocerol diesters are composed of a mixture of long chain β-diols that are esterified by multimethyl-branched fatty acids. Depending on the asymmetric centers bearing the methyl branches (D or L series), the fatty acids are called mycocerosic or phthioceranic acids, respectively, and the corresponding complex lipids are named dimycocerosates of phthiocerol (DIMs) or diphthioceranates of phthiocerol (DIPs) (*1*). The phenolic glycolipids (PGLs) consist of a lipid core similar to those of DIMs or DIPs but ω-terminated by an aromatic nucleus that is glycosylated by type- or species-specific mono-, tri-, or tetrasaccharide. Several lines of evidences suggest that PGLs are involved in the pathogenesis of mycobacterial infections. PGL-1 from *M. leprae* inhibits the proliferation of T lymphocytes after stimulation with concanavalin A (*2*). Moreover, PGL-1 seems to be associated with resistance to intracellular killing by macrophages (*3*) and promotes phagocytosis of *M. leprae* by macrophages and Schwann cells by binding to complement component C3 or laminin α2 chain, respectively (*4**,**5*). Similarly, PGLs produced by a subset of *M. tuberculosis* isolates inhibit the host Th1-type T-cell and cytokine response (*6*). All *M. tuberculosis* strains tested that produce PGLs belong to the W-Beijing family and show a "hypervirulent" phenotype, in comparison with the clinical isolate *M. tuberculosis* CDC1551 and the laboratory strain *M. tuberculosis* H37Rv in the murine model (*6*) and rabbit model of meningitis (*7*).

Previous study identified the involvement of the gene *pks15/1* in the biosynthesis of PGLs; disruption of this gene generated a PGL-deficient mutant (*8*). Sequence alignment of the *pks15/1* gene, when compared to the non–PGL-producing strains, *M. tuberculosis* H37Rv, Erdman, Mt103, and CDC1551, that contain 2 open reading frames [*pks1* (Rv2946c) and *pks1*5 (Rv2947c)], showed a 7-bp insertion in PGL-producing strains *M. tuberculosis* strain 210, belonging to the W-Beijing family, and *M. canetti*, whereas *M. bovis* and *M. bovis* BCG contained only a guanine insertion. This 7-bp or 1-bp insertion causes a frameshift mutation in the *pks1*5, resulting in an intact *pks15/1* with additional codons (*8*). Similar results have been shown in other W-Beijing strains, *M. tuberculosis* HN878, W4, and W10, which contain the 7-bp insertion and produce PGLs (*6*).

In Thailand, the Beijing genotype is the predominant genotype among tuberculosis (TB) patients, particularly in patients with TB meningitis (unpub. data), which suggests recent transmission of this genotype in the country. Similarly, the Beijing genotype has been found frequently in Asia (*9**–**11*). Previous studies have shown that the *M. tuberculosis* strains belonging to this genotype contain an intact *pks15/1* and can produce PGLs that associated with the hypervirulent phenotype (*6**,**7*). The goal of our study was to determine whether the hypervirulence of the W-Beijing strains due to the ability to produce PGLs is unique among this family by investigating the *pks15/1* gene of the Beijing strains compared to other strains that can cause diseases similar to those caused by Beijing strains.

## The Study

One hundred forty-seven clinical isolates of *M. tuberculosis* were obtained from the Molecular Mycobacteriology Laboratory, Department of Microbiology, Faculty of Medicine, Siriraj Hospital, Mahidol University, Thailand, and the T-2 project from 1997 to 2001 (Table). These strains were isolated from 74 cerebrospinal fluid (CSF) samples and 73 sputum samples from 147 different patients. DNA from these isolates was isolated by an enzymatic method and submitted for genotyping by performing the IS*6110* restriction fragment length polymorphism with the standard method (*12*) and for sequencing the *pks15/1* region (*8*).

**Table:** Number of *Mycobacterium tuberculosis* genotypes and strains containing an intact pks15/1*

Genotype	No. strains isolated from CSF	No. CSF strains containing intact *pks15/1* (%)	No. strains isolated from sputum	No. sputum strains containing intact *pks15/1* (%)
Beijing	42	41 (97.6)	31	30 (96.8)
Single-banded	10	8 (80.0)	10	9 (90.0)
2–5 bands	5	4 (80.0)	11	10 (90.9)
Nonthaburi	4	4 (100)	8	8 (100)
Heterogeneous with >5 bands	13	8 (61.5)	13	5 (38.5)
Total	74	65 (87.8)	73	62 (84.9)

*CSF, cerebrospinal fluid.

**Figure 1:**
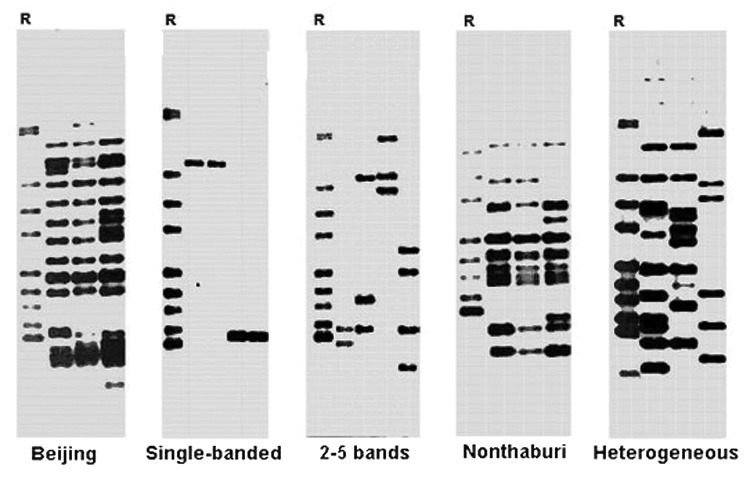
IS*6110* hybridization patterns of each *Mycobacterium tuberculosis* genotype. R indicates the *M. tuberculosis* Mt 14323 strain used as the positive control for IS*6110* typing.

**Figure 2:**
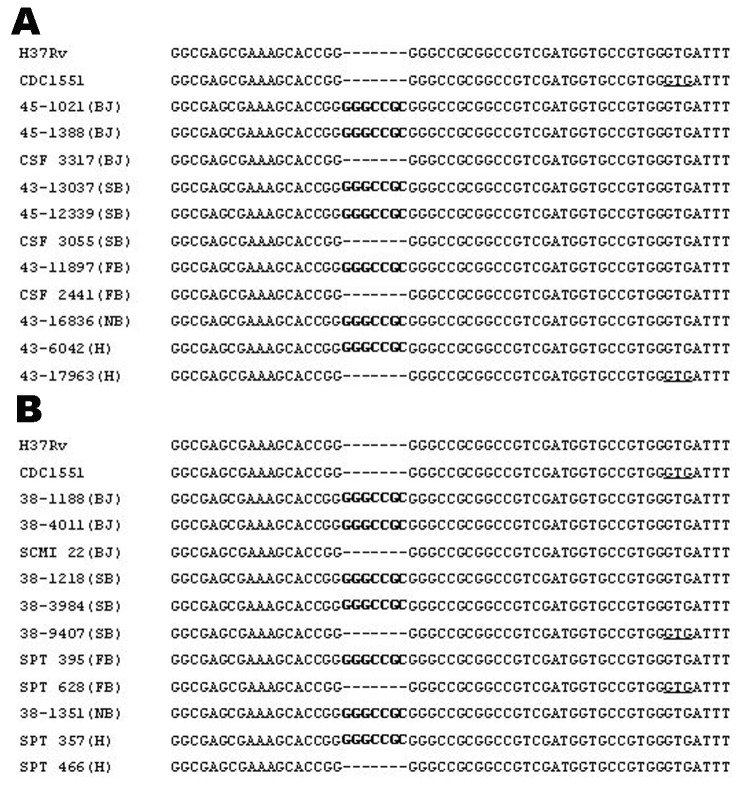
Sequence alignment of region corresponding to the 3´ portion of *pks15* and 5´ portion of *pks1* in various *Mycobacterium tuberculosis* genotypes. A) *M. tuberculosis* strains isolated from cerebrospinal fluid. B) *M. tuberculosis* strains isolated from sputum. Letters in brackets refer to IS*6110* restriction fragment length polymorphism patterns: BJ, Beijing; SB, single banded; FB, 2–5 bands; NB, Nonthaburi; H, heterogeneous. The 7-bp insertion is shown in **boldface**, and the start codon of the *pks1* gene is underlined.

Using the genotyping results, we categorized *M. tuberculosis* isolates into Beijing, single-banded, few-banded (2–5 bands), Nonthaburi, and heterogeneous with >5 bands (Table and Figure 1), as recently reported (*13**,**14*). All *M. tuberculosis* genotypes were sequenced around the junction of *pks1*5 and *pks1* (corresponding to the *M. tuberculosis* H37Rv sequence) to determine whether they contained an intact *pks15/1* or separated *pks1*5 and *pks1*. Unexpectedly, the results showed that the 7-bp insertion of *pks1*5 that causes a frameshift mutation resulting in an intact *pks15/1* was found in most strains of all genotypes, except the heterogeneous group with >5 bands (Table and Figure 2).

## Conclusions

The intact *pks15/1* has been shown to be responsible for the production of phenolic glycolipids and is seemingly found in *M. tuberculosis* W-Beijing family, but it was not found in *M. tuberculosis* CDC1551 and H37Rv (*8*). Previous studies suggested that PGLs produced by the *M. tuberculosis* W-Beijing family were associated with the hypervirulent phenotype by inhibiting the innate immune response (*6**,**7*). The intact *pks15/1* has also been shown to be nonpolymorphic in the W-Beijing family; it was found in all 102 W-Beijing strains tested (*15*). From this observation, we hypothesized that if the ability to produce PGLs is among the factors that make this family more virulent than others, the intact *pks15/1* should be absent in strains other than the W-Beijing family. Our results showed that the 7-bp insertion of the *pks15/1* was not only present in the W-Beijing family but also in other *M. tuberculosis* genotypes. Although almost all Beijing strains contain the intact *pks15/1* (≈97%), 38.5%–100% of strains of other genotypes also contain it. These strains could, therefore, produce PGLs and cause both pulmonary and disseminated diseases as the W-Beijing strains do.

Our results showed no significant difference in the percentage of *M. tuberculosis* isolates with an intact *pks15/1* gene between CSF isolates (65 [87.8%] of 74) and sputum isolates (62 [84.9%] of 73). The hypothesis that the hypervirulence of the W-Beijing family is solely attributable to *pks15/1* is still inconclusive. This family may have only recently been transmitted globally and may have had more chances to cause infections and disease than other families. Although PGLs are involved in the hypervirulence of the PGL-producing strains, they are not a unique characteristic of the W-Beijing family. If W-Beijing strains are more virulent than others, other virulence determinants besides PGLs must be responsible for the hypervirulent phenotype.
